# Bidirectional Associations Between Anxiety and Gastrointestinal Disorders: A Retrospective Cohort Study Using the TriNetX Database

**DOI:** 10.7759/cureus.102747

**Published:** 2026-01-31

**Authors:** Zachary Li, Leah Morgan, Taylor M Gong, Manasa Mula, Eduardo Espiridion

**Affiliations:** 1 Psychiatry, Drexel University College of Medicine, Wyomissing, USA; 2 Psychiatry, Reading Hospital, Tower Health, West Reading, USA

**Keywords:** anxiety disorders, gastrointestinal disorders, gut-brain axis, gut dysbiosis, inflammatory bowel disease, irritable bowel syndrome, mental health

## Abstract

Introduction: Irritable bowel syndrome (IBS) and inflammatory bowel disease (IBD) are common gastrointestinal (GI) disorders often linked with anxiety and depression. IBS is defined by recurrent abdominal pain and altered bowel habits without a detectable organic disease, whereas IBD involves chronic, relapsing intestinal inflammation. Bidirectional communication through the gut-brain axis, gut dysbiosis, neuroimmune interactions, and shared genetic susceptibility may underlie these associations. This study aims to quantify and compare the bidirectional associations between anxiety and IBS or IBD using a large, real-world multicenter dataset.

Methods: A retrospective cohort study was conducted using TriNetX, a global network of de-identified electronic health records from over 100 healthcare organizations. Adults diagnosed with anxiety disorders, IBS, and IBD between January 1, 2021, and January 1, 2024, were identified along with matched controls. Patients with prior psychiatric disorders, GI conditions, recent GI infections, or relevant medication use were excluded. Propensity score matching balanced cohorts by age, sex, race, ethnicity, smoking status, and history of depression. Outcomes included incident IBS or IBD following anxiety and incident anxiety following IBS or IBD assessed over a follow-up period of five years post-index. Risk ratios, risk differences, and odds ratios with 95% confidence intervals were calculated.

Results: Among matched cohorts, 6,724 individuals (1.1%) with anxiety developed IBS compared with 2,457 (0.4%) in controls (RR 2.737, 95% CI 2.613-2.866; p < 0.001). For IBD, 1,412 patients (0.2%) with anxiety developed the condition versus 908 (0.2%) in controls (RR 1.555, 95% CI 1.431-1.690; p < 0.001). Reciprocally, 8,334 patients (6.6%) with IBS developed anxiety compared with 3,770 (3.0%) in controls (RR 2.211, 95% CI 2.129-2.295; p < 0.001). Among patients with IBD, 2,354 (2.6%) developed anxiety versus 2,383 (2.7%) in controls (RR 0.988, 95% CI 0.934-1.045; p = 0.669).

Conclusion: Anxiety and IBS exhibit strong bidirectional associations, whereas links between anxiety and IBD are weaker and inconsistent. These findings highlight the roles of the gut-brain axis and neuroimmune pathways in these conditions. Screening for psychiatric comorbidities and integrating GI and mental health care, including gut-brain-targeted interventions, may improve outcomes for patients suffering from these conditions.

## Introduction

Irritable bowel syndrome (IBS) is one of the most common functional gastrointestinal (GI) disorders, affecting an estimated 5-10% of the global population, with higher prevalence among women and notable regional variation [1,2]. IBS is characterized by recurrent abdominal pain associated with altered bowel habits in the absence of a detectable organic disease [1]. Common symptoms include abdominal pain, bloating, diarrhea, constipation, or mixed bowel patterns, in addition to extraintestinal symptoms such as headaches and fatigue. The pathophysiology of IBS is multifactorial, involving dysregulation of the gut-brain axis (GBA), visceral hypersensitivity, dysmotility, immune activation, changes in the gut microbiota, and psychosocial factors [2,3]. Current management focuses on lifestyle and dietary modifications along with pharmacologic therapy tailored to the IBS subtype. 

Inflammatory bowel disease (IBD), encompassing Crohn’s disease and ulcerative colitis, is characterized by chronic, relapsing inflammation of the GI tract. Although its exact cause remains unclear, IBD is thought to result from an inappropriate immune response to intestinal microbiota in genetically susceptible individuals, influenced by environmental factors such as smoking [4,5]. Both subtypes are associated with significant morbidity, including reduced quality of life and increased colorectal cancer risk. Current management of IBD centers around aminosalicylates, corticosteroids, and immunomodulators such as tumor necrosis factor inhibitors [6].

The GBA is a bidirectional communication network linking the central nervous system (CNS) and the GI tract through neural, immune, and hormonal pathways [7,8]. Disruptions in the GBA have been associated with GI and psychiatric conditions, including IBS, IBD, and anxiety disorders [1,4]. IBS is thought to reflect functional dysregulation within the GBA, whereas IBD involves immune-mediated inflammatory disturbances that may also interact with brain signaling [9]. Anxiety and depression may not only arise as secondary consequences but also act as risk factors for the subsequent development of GI disease [5,7]. Longitudinal studies show that anxiety or heightened stress can precede GI symptoms, increasing the risk of IBS and IBD [5,10]. Conversely, GI symptoms and chronic intestinal inflammation can precede anxiety, highlighting bidirectional gut-brain interactions in psychiatric and GI disease [1,11]. 

This framework underscores the importance of studying gut-brain interactions across both functional and inflammatory contexts, with the potential to inform novel treatments for GI and psychiatric disorders. Despite growing recognition of these links, large-scale, real-world data describing the strength and direction of these associations remain limited. The TriNetX Global Collaborative Network provides an opportunity to examine these relationships across diverse health systems. This study aims to quantify and compare the bidirectional association between anxiety and both IBS and IBD using multicenter electronic health record data.

## Materials and methods

This retrospective cohort study used TriNetX, a multi-national federated research network that aggregates de-identified electronic health records from over 100 healthcare organizations, the majority of which are based in the United States. The data used in this study were collected between January 1, 2021, and January 1, 2024, using the TriNetX Global Collaborative Network, which provides access to electronic medical records (diagnoses, laboratory values, procedures, genomic information, medications) from approximately 162 healthcare organizations. Institutional Review Board (IRB) approval was not required for this study. The index event was defined as the first recorded occurrence of the relevant diagnostic code, and patients whose index event occurred more than 20 years before analysis were excluded. Diagnoses were identified using International Classification of Diseases, Tenth Revision (ICD-10) codes, a classification system maintained by the World Health Organization for disease classification. Medication exposures were identified with National Library of Medicine (NLM) codes, Anatomical Therapeutic Chemical Classification System (ATC) codes, and Veterans Affairs (VA) classifications.

Five cohorts were created to examine the associations between anxiety disorders and GI conditions, specifically IBS and IBD. Three separate temporal analyses were created in order to analyze the risk. For analyses evaluating the risk of IBS or IBD following anxiety, adults aged 18 years or older with a diagnosis of an anxiety disorder were identified, along with a control cohort consisting of adults with encounters for routine health examinations and no diagnosis of anxiety. Individuals with a prior diagnosis of IBS or IBD were excluded.

For reciprocal analyses evaluating the risk of anxiety following GI disease, separate cohorts of adults with IBS or IBD and no prior history of anxiety or the alternate GI condition were identified. A corresponding control cohort included adults with routine health examinations and no history of anxiety, IBS, or IBD.

Across all cohorts, uniform exclusion criteria were applied to reduce confounding with pharmacologic, psychiatric, and GI factors. Patients were excluded if they had prior use of antidepressants, benzodiazepine derivatives, or other sedative-hypnotics, antipsychotics, and/or opioids, as these medications may independently influence mood and GI function [12,13]. Additional exclusions included a history of bipolar disorder, schizophrenia, schizotypal disorder, delusional disorder, other non-mood psychotic disorders, celiac disease, malignant neoplasm of the rectosigmoid junction, and intestinal infectious disease within the preceding six months, given the potential of these conditions to affect both GI and psychiatric outcomes [14,15]. For analyses involving IBS and IBD as exposures, patients with a prior diagnosis of IBS were excluded from the IBD cohort, and vice versa, to preserve diagnostic clarity. Lastly, individuals with pre-existing diagnoses of the outcome of interest were excluded to ensure temporal clarity. A complete list of diagnostic codes and exclusion criteria used for cohort construction is provided in Table 1.

**Table 1:** Diagnostic Codes and Criteria Used for Cohort Construction The table lists categories, their associated diagnostic and medication codes, and the rationale for inclusion or exclusion in the analyses. Diagnoses and medication exposures were identified using International Classification of Diseases, Tenth Revision (ICD-10) codes, National Library of Medicine (NLM) codes, Anatomical Therapeutic Chemical Classification System (ATC) codes, and Veterans Affairs (VA) classifications.

Category	Codes Used	Rationale
Anxiety disorders	ICD-10: F41.9	Cohort inclusion for bidirectional analysis
Irritable Bowel Syndrome	ICD-10: K58	Cohort inclusion for bidirectional analysis
Inflammatory Bowel Disease	ICD-10: K50–K51	Cohort inclusion for bidirectional analysis
Psychiatric Exclusions	ICD-10: F31, F20–F29	Exclusion criteria: confounders affecting anxiety/GI outcomes
GI Condition Exclusions	ICD-10: K90.0, C19	Exclusion criteria: prevent confounding with major GI disease
Infectious GI disease	ICD-10: A00–A09	Exclusion criteria: prevent post-infectious IBS/anxiety bias
Medication Exclusions	Antidepressants (NLM:VA:CN600) Benzodiazepines/sedatives (NLM:VA:CN302) Antipsychotics (ATC:N05A) Opioids (NLM:VA:CN101)	Exclusion criteria: avoid pharmacologic confounding
Prior diagnosis of outcome	Outcome-specific ICD-10 codes	Exclusion criteria: Ensure temporal clarity

Propensity score matching was performed using a nearest-neighbor algorithm with a caliper of 0.1 to balance each exposure cohort with its respective control on age, sex, race, ethnicity, smoking status, and history of depressive episode. After matching, each analysis yielded two balanced cohorts for outcome assessment. For analyses in which anxiety was the exposure, the primary outcomes were new diagnoses of IBS or IBD. For analyses in which IBS or IBD served as the exposure, the primary outcome was a new diagnosis of an anxiety disorder. Outcomes were assessed over a five-year follow-up period beginning one day after the index event. Risk analyses were conducted using the TriNetX Analytics platform and are reported as risk ratios (RRs), risk differences (RDs), and odds ratios (ORs) with corresponding 95% confidence intervals (CIs).

## Results

This study evaluated the associations between anxiety and gastrointestinal conditions using separate longitudinal analyses with propensity-matched cohorts derived from the TriNetX Global Collaborative Network. 

Prior to matching, the anxiety cohort consisted of N = 636,712 patients with no history of IBS or IBD, and the control cohort consisted of N = 7,890,540 individuals with no diagnoses of anxiety, IBS, or IBD. For the reciprocal analyses, the IBS cohort included N = 141,752 individuals with no prior IBD or anxiety, the IBD cohort included N = 97,228 individuals with no prior IBS or anxiety, and the corresponding control group included N = 8,042,284 individuals without anxiety or gastrointestinal disease. Following matching, each analysis yielded balanced cohorts of equal size: 601,315 patients in the anxiety versus control analysis, 127,205 in the IBS versus control analysis, and 89,509 patients in the IBD versus control analysis. Baseline demographic and clinical characteristics of the propensity-matched cohorts are summarized in Table 2. Cohort sizes before and after propensity score matching for each analytic comparison are shown in Table 3. 

**Table 2:** Demographics and Clinical Characteristics of Matched Cohorts The table displays baseline demographic information for the propensity-score matched cohorts used in analyses of the relationship between anxiety and gastrointestinal disorders. Covariate balance was achieved, with standardized mean differences (SMDs) below 0.01 in all analyses, except for a small residual imbalance in the ‘Unknown race’ category in the IBD to anxiety and IBS to anxiety analyses, which showed SMDs of 0.013 and 0.006, respectively. Patients categorized as unknown sex, Native Hawaiian or Other Pacific Islander, and American Indian or Alaska Native were not included as matching covariates due to limitations of the TriNetX platform when performing propensity score matching in large cohorts. These patients were included in the matched cohorts, resulting in demographic subgroup totals that may not sum to the full matched sample size. IBS: Irritable bowel syndrome; IBD: inflammatory bowel disease

Characteristic	Analysis 1: Anxiety → IBS + IBD Cohorts	Analysis 2: IBS → Anxiety Cohorts	Analysis 3: IBD → Anxiety Cohorts
Sample Size (Matched)	601,315	127,205	89,509
Age, mean ± SD	42.7 ± 18.2	49.2 ± 18.3	47.9 ± 18.1
Sex			
Female	398,287 (66.2%)	87,261 (68.6%)	43,828 (49.0%)
Male	202,659 (33.7%)	39,889 (31.4%)	45,624 (51.0%)
Race			
White	385,873 (64.2%)	75,190 (59.1%)	52,260 (58.8%)
Black or African American	68,713 (11.4%)	8,387 (6.6%)	6,500 (7.3%)
Asian	20,325 (3.4%)	5,003 (3.9%)	2,994 (3.3%)
Other race	25,956 (4.3%)	6,625 (5.2%)	2,932 (3.3%)
Unknown race	95,631 (15.9%)	31,405 (24.7%)	23,598 (26.4)
Ethnicity			
Hispanic or Latino	48,437 (8.1%)	4,978 (3.9%)	3,050 (3.4%)
Not Hispanic or Latino	385,522 (64.1%)	66,081 (51.9%)	48,456 (54.1%)
Unknown ethnicity	167,356 (27.8%)	56,155 (44.1%)	38,003 (42.5%)

**Table 3:** Cohort Sizes Before and after Propensity Score Matching The table displays the number of participants in each exposure and control group prior to and after propensity score matching. Pre-match cohort sizes are reported as absolute counts with percentages representing the proportion of the total eligible study population for each analysis. Final matched cohorts are reported as absolute counts and percentages of the total matched population. IBS: Irritable bowel syndrome; IBD: inflammatory bowel disease

Comparison	Exposure Pre-Match	Control Pre-Match	Final Matched Exposure	Final Matched Control
Analysis 1: Anxiety → IBS + IBD	601,342 (7.38%)	7,544,665 (92.62%)	601,315 (50%)	601,315 (50%)
Analysis 2: IBS → Anxiety	127,205 (1.61%)	7,759,661 (98.39%)	127,205 (50%)	127,205 (50%)
Analysis 3: IBD → Anxiety	89,509 (1.15%)	7,693,156 (98.85%)	89,509 (50%)	89,509 (50%)

Among patients with anxiety and matched controls, 6,724 individuals (1.1%) in the anxiety cohort developed IBS compared with 2,457 individuals (0.4%) in the control cohort. The RD was 0.007 (95% CI: 0.007, 0.007, p < 0.001), corresponding to a RR of 2.737 (95% CI: 2.613, 2.866) and an OR of 2.756 (95% CI: 2.631, 2.887). For IBD, 1,412 patients (0.2%) in the anxiety cohort developed the condition compared with 908 patients (0.2%) in the control cohort. The RD was 0.001 (95% CI: 0.001, 0.001, p < 0.001), with a RR of 1.555 (95% CI: 1.431, 1.690) and an OR of 1.556 (95% CI: 1.432, 1.692).

In the reciprocal analysis of IBS and anxiety, 8,334 patients (6.6%) in the IBS cohort developed anxiety compared with 3,770 patients (3.0%) in the control group. The RD was 0.036 (95% CI: 0.034, 0.038, p < 0.001), with a RR of 2.211 (95% CI: 2.129, 2.295) and an OR of 2.295 (95% CI: 2.207, 2.387). For the IBD analysis, 2,354 patients (2.6%) in the IBD cohort developed anxiety compared with 2,383 patients (2.7%) in the control cohort. The RD was -0.000 (95% CI: -0.002, 0.001, p = 0.669), with a RR of 0.988 (95% CI: 0.934, 1.045) and an OR of 0.988 (95% CI: 0.932, 1.046). Risk estimates for all primary analyses, including RDs and RRs, are summarized in Table 4. 

**Table 4:** Bidirectional Risk of Anxiety and Gastrointestinal Disease After Propensity Score Matching The table presents outcome event counts, absolute risks, risk differences, risk ratios (RRs), and p-values for propensity score–matched cohorts evaluating the risk of developing IBS or IBD following a diagnosis of anxiety and the risk of developing anxiety following a diagnosis of IBS or IBD. IBS: Irritable bowel syndrome; IBD: inflammatory bowel disease

Comparison	Exposure Events	Control Events	Exposure Risk (%)	Control Risk (%)	Risk Difference (95% CI)	Risk Ratio (95% CI)	P-value
Anxiety → IBS	6,724	2,457	1.10%	0.40%	0.007 (0.007–0.007)	2.737 (2.613–2.866)	<0.001
Anxiety → IBD	1,412	908	0.20%	0.20%	0.001 (0.001–0.001)	1.555 (1.431–1.690)	<0.001
IBS → Anxiety	8,334	3,770	6.60%	3.00%	0.036 (0.034–0.038)	2.211 (2.129–2.295)	<0.001
IBD → Anxiety	2,354	2,383	2.60%	2.70%	-0.000 (-0.002–0.001)	0.988 (0.934–1.045)	0.669

## Discussion

This large, multi-institutional cohort study found strong bidirectional associations between anxiety and IBS in separate temporal analyses, and weaker, inconsistent associations between anxiety and IBD. After propensity score matching across demographic and clinical variables, patients with a diagnosis of anxiety demonstrated a significantly higher incidence of both subsequent IBS and IBD diagnoses compared to controls over a five-year follow-up period. In the reciprocal analysis, patients with IBS had a significantly higher incidence of a subsequent diagnosis of anxiety, whereas patients with IBD showed no significant difference in anxiety incidence.

The consistent relationship between anxiety and IBS observed in this study is consistent with prior research. Meta-analyses have previously shown that IBS is associated with increased anxiety compared to healthy controls, and that anxiety and heightened stress sensitivity are significant risk factors for the development and exacerbation of IBS, reinforcing that both these disorders are sensitive to disruptions within the GBA [1,16]. To explain these associations, prior research has identified neurobiological and psychosocial pathways that link emotional regulation with GI function.

The enteric nervous system (ENS) comprises interconnected neural networks that are responsible for regulating gut motility, secretion, and sensation. The ENS interacts closely with the gut microbiome and communicates with the CNS through vagal and sympathetic pathways. Dysregulation of these circuits through psychosocial or gut-derived stress has been implicated in both IBS and anxiety [1]. For example, studies demonstrate that stress, microbial dysbiosis, and alterations in neurotransmitter signaling can influence both gut function and emotional processing [17,18]. 

Genetic studies further support shared biological pathways. Genes such as CADM2 and NCAM1 are implicated in synaptic organization and ENS development and thus associated with anxiety, depression, and IBS [19,20]. Neuroimaging research also shows that IBS patients exhibit differences such as decreased volume in brain regions related to emotional regulation and interoception, including the prefrontal cortex, anterior insula, and hippocampus [21]. Collectively, these data reinforce the concept of IBS as a disorder deeply rooted in gut-brain-microbiome interactions.

However, the associations between anxiety and IBD are weaker and asymmetric, with higher rates of anxiety and depression observed particularly during disease flares [7]. This relationship appears less connected to disease onset, which is a key distinction when compared with IBS. Mechanistically, IBD-related inflammation may influence mood through cytokine-mediated neuroimmune signaling, vagal and nociceptive pathways, microbial metabolites, and increased blood-brain barrier permeability [8,11]. Microglial activation and inflammatory changes in brain regions implicated in anxiety, such as the anterior cingulate cortex, have also been described. However, these mechanisms likely operate selectively depending on disease severity, intestinal location, and individual inflammatory profiles [8]. 

Notably, several studies suggest that psychological distress may precede or promote IBD onset more strongly than IBD promotes future anxiety, which aligns with our study results [5]. Chronic stress may potentiate mucosal inflammation through hypothalamic-pituitary-adrenal axis dysregulation, neuroimmune activation, and enteric glial responses [22]. Variability across studies assessing the influence of IBD on the development of anxiety may reflect heterogeneity in disease activity, treatment intensity, and timing of psychiatric assessment. Although our study did not identify a significant association between IBD and subsequent anxiety, these findings are consistent with prior work demonstrating that psychological symptoms nonetheless influence IBD activity [7,18].

Comparison across disorders indicates that the associations in both temporal directions between anxiety and IBS are substantially stronger than those between anxiety and IBD. Several mechanisms may contribute to this distinction. IBS involves heightened sensitivity within the GBA, strong psychosocial modulation, and substantial genetic and neurobiological overlap with anxiety disorders. In contrast, IBD is driven primarily by immune-mediated pathology, with psychological factors exerting more influence on disease course than on disease initiation. These mechanistic differences are further reflected in the way patients perceive their illness, with prior research showing that IBS patients often have more negative illness perceptions and greater concern about their condition compared with patients with IBD. This may, in turn, heighten anxiety responses and contribute to symptom amplification [23]. Together, these differences underscore the importance of tailoring psychological assessment and intervention strategies to the underlying pathophysiology of each condition.

Understanding the relationship between GI disorders and common neuropsychiatric comorbidities highlights the importance of bidirectional screening, both for neuropsychiatric disorders in patients receiving care for GI conditions and for the development of GI disorders in patients with neuropsychiatric conditions. Furthermore, recognizing the mechanisms linking neuropsychiatric disorders, such as anxiety, with GI disorders supports treatments that address both domains. These considerations underscore the need for integrated, multidisciplinary care that targets both GI and psychiatric symptoms.

Although our analysis did not evaluate therapeutic outcomes, the observed associations have implications for integrated clinical management. For example, prior research has shown that gut-brain neuromodulators such as TCAs, SSRIs, and SNRIs have efficacy in reducing global and abdominal IBS symptoms and improving anxiety [24]. Similar agents may improve IBD outcomes by reducing inflammation, modulating pain pathways, and altering the gut microbiota [25,26]. Recognizing anxiety as both a risk factor for and consequence of GI symptoms supports early identification and multidisciplinary care. Anxiety can worsen GI symptoms once established, reinforcing a feedback loop that may exacerbate disease burden. Collaboration between gastroenterologists, psychiatrists, and behavioral health specialists may therefore help break this cycle, particularly in individuals with subclinical anxiety preceding GI symptom onset.

This study has several limitations related to its retrospective design and use of electronic health record data. First, due to the observational and retrospective nature of this study, we are not able to draw any causal relationships despite the associations between anxiety and GI disorders identified in this study. Secondly, the identification of IBS, IBD, and anxiety relied on ICD-10 diagnostic codes. Misclassification is possible given that the coding and diagnostic accuracy may differ across health care organizations and providers associated with the TriNetX platform. In addition, reliance on these codes may underestimate the true prevalence of anxiety and GI disease, as many patients with subclinical symptoms or those treated outside formal psychiatric settings may not receive coded diagnoses. The use of diagnostic codes also limits the ability to assess disease severity, preventing distinctions between mild and severe disease. Moreover, medication exposure may be under-captured if prescriptions were written outside participating health systems. 

Third, although propensity score matching was used to reduce confounding, residual confounding from unmeasured variables remains possible. TriNetX lacks consistent data on factors such as socioeconomic status, dietary habits, stress levels, and medication adherence, all of which may influence both anxiety and GI outcomes. Although we excluded patients with documented exposure to antidepressants, benzodiazepines, antipsychotics, and opioids, residual confounding from unmeasured or other medication exposure such as corticosteroids, may have influenced both GI and psychiatric outcomes. In addition, it was not possible to determine whether anxiety symptoms occurred in relation to GI disease flares or changes in disease activity; only the presence of a diagnosis could be recorded. Furthermore, the number, timing, and duration of GI or psychiatric symptom episodes could not be assessed. Finally, the extensive exclusion criteria required to minimize pharmacologic, psychiatric, and GI confounding may limit generalizability. Patients with complex psychiatric histories, recent GI infections, or exposure to psychotropic or opioid medications were excluded, and these populations may experience different patterns of gut-brain interactions than those represented in this analysis. Nonetheless, our study supports important clinical relationships between anxiety, IBS, and IBD.

Future research should aim to overcome the limitations associated with this retrospective study to better uncover the mechanisms underlying the association demonstrated in this study and the broader gut-brain relationship. Prospective, longitudinal cohorts using standardized diagnostic criteria for anxiety, IBS, and IBD are needed to establish temporal relationships between these conditions. In addition, interventional randomized controlled trials could be beneficial in identifying multimodal treatment strategies to address these disease processes. For example, whether treating anxiety through modalities like CBT or pharmacological approaches could reduce IBS or IBD risk or symptom severity, and conversely, whether optimizing GI disease control improves psychiatric outcomes. Lastly, future studies could combine health record data with analysis of microbiome sequencing and neuroendocrine markers to identify key biological intermediates that link anxiety and GI disease. More broadly, these intermediates could be compared with those in other GI disorders with psychiatric comorbidities to better elucidate the complex gut-brain axis. Future large-scale cohort studies should aim to clarify the magnitude of psychiatric-to-GI disease risk as well as the converse relationship, and further explore any differences found in the magnitude of these associations.

## Conclusions

In this large, multi-institutional retrospective cohort study, anxiety and IBS demonstrated strong bidirectional associations based on two separate analyses, while the relationship between anxiety and IBD was weaker and asymmetric. These findings highlight important differences in how functional and inflammatory GI disorders interact with psychological symptoms and the GBA. Although mechanistic pathways likely differ across conditions, the consistent association between anxiety and GI outcomes underscores the clinical importance of recognizing and addressing psychological symptoms in GI care, as well as remaining vigilant for GI symptoms in patients presenting with psychiatric disorders to improve overall outcomes.

## References

[1] Mayer EA, Ryu HJ, Bhatt RR (2023). The neurobiology of irritable bowel syndrome. Mol Psychiatry.

[2] Ford AC, Sperber AD, Corsetti M, Camilleri M (2020). Irritable bowel syndrome. Lancet.

[3] Black CJ, Drossman DA, Talley NJ, Ruddy J, Ford AC (2020). Functional gastrointestinal disorders: advances in understanding and management. Lancet.

[4] Bisgaard TH, Allin KH, Keefer L, Ananthakrishnan AN, Jess T (2022). Depression and anxiety in inflammatory bowel disease: epidemiology, mechanisms and treatment. Nat Rev Gastroenterol Hepatol.

[5] Bisgaard TH, Allin KH, Elmahdi R, Jess T (2023). The bidirectional risk of inflammatory bowel disease and anxiety or depression: a systematic review and meta-analysis. Gen Hosp Psychiatry.

[6] Cai Z, Wang S, Li J (2021). Treatment of inflammatory bowel disease: a comprehensive review. Front Med (Lausanne).

[7] Fairbrass KM, Lovatt J, Barberio B, Yuan Y, Gracie DJ, Ford AC (2022). Bidirectional brain-gut axis effects influence mood and prognosis in IBD: a systematic review and meta-analysis. Gut.

[8] Masanetz RK, Winkler J, Winner B, Günther C, Süß P (2022). The gut-immune-brain axis: an important route for neuropsychiatric morbidity in inflammatory bowel disease. Int J Mol Sci.

[9] Gracie DJ, Guthrie EA, Hamlin PJ, Ford AC (2018). Bi-directionality of brain-gut interactions in patients with inflammatory bowel disease. Gastroenterology.

[10] Sibelli A, Chalder T, Everitt H, Workman P, Windgassen S, Moss-Morris R (2016). A systematic review with meta-analysis of the role of anxiety and depression in irritable bowel syndrome onset. Psychol Med.

[11] Yuan X, Chen B, Duan Z (2021). Depression and anxiety in patients with active ulcerative colitis: crosstalk of gut microbiota, metabolomics and proteomics. Gut Microbes.

[12] Kwak N, Lee H, Kim BK, Yu YM, Kang HY (2024). Association between selective serotonin reuptake inhibitor use and developing irritable bowel syndrome through retrospective analysis. J Gastroenterol Hepatol.

[13] Bernstein CN, Fisk JD, Walld R (2022). Use of benzodiazepines and Z-drugs in inflammatory bowel disease. Am J Gastroenterol.

[14] Lee YT, Hu LY, Shen CC (2015). Risk of psychiatric disorders following irritable bowel syndrome: a nationwide population-based cohort study. PLoS One.

[15] Schwille-Kiuntke J, Enck P, Polster AV, Gaile M, Kremsner PG, Zanger P (2015). Postinfectious irritable bowel syndrome after travelers' diarrhea--a cohort study. Neurogastroenterol Motil.

[16] Zamani M, Alizadeh-Tabari S, Zamani V (2019). Systematic review with meta-analysis: the prevalence of anxiety and depression in patients with irritable bowel syndrome. Aliment Pharmacol Ther.

[17] Person H, Keefer L (2021). Psychological comorbidity in gastrointestinal diseases: update on the brain-gut-microbiome axis. Prog Neuropsychopharmacol Biol Psychiatry.

[18] Peppas S, Pansieri C, Piovani D (2021). The brain-gut axis: psychological functioning and inflammatory bowel diseases. J Clin Med.

[19] Morris J, Bailey ME, Baldassarre D (2019). Genetic variation in CADM2 as a link between psychological traits and obesity. Sci Rep.

[20] Mota NR, Rovaris DL, Kappel DB (2015). NCAM1-TTC12-ANKK1-DRD2 gene cluster and the clinical and genetic heterogeneity of adults with ADHD. Am J Med Genet B Neuropsychiatr Genet.

[21] Li Z, Ma Q, Deng Y (2024). Irritable bowel syndrome is associated with brain health by neuroimaging, behavioral, biochemical, and genetic analyses. Biol Psychiatry.

[22] Schneider KM, Blank N, Alvarez Y (2023). The enteric nervous system relays psychological stress to intestinal inflammation. Cell.

[23] MacKrill K, Bass C, Petrie K (2025). Comparing illness perceptions in inflammatory bowel disease and irritable bowel syndrome. J Psychosom Res.

[24] Khasawneh M, Mokhtare M, Moayyedi P, Black CJ, Ford AC (2025). Efficacy of gut-brain neuromodulators in irritable bowel syndrome: an updated systematic review and meta-analysis. Lancet Gastroenterol Hepatol.

[25] Di Vincenzo F, D'Onofrio AM, Del Gaudio A (2025). Psychopharmacological therapy positively modulates disease activity in inflammatory bowel disease: a systematic review. Int J Mol Sci.

[26] Xu F, Xie Q, Kuang W, Dong Z (2023). Interactions between antidepressants and intestinal microbiota. Neurotherapeutics.

